# Differential Diagnosis and Hospital Emergency Management for Fastlane Treatment of Central Nervous System Infection Under the COVID-19 Epidemic in Changsha, China

**DOI:** 10.3389/fneur.2020.555202

**Published:** 2020-10-20

**Authors:** Haojun Yang, Yunfang Chi, Zhuohui Chen, Yishu Fan, Haiyue Wu, Xinhang Hu, Tong Wu, Bo Xiao, Mengqi Zhang

**Affiliations:** ^1^Department of Neurology, Xiangya Hospital of Central South University, Changsha, China; ^2^Laizhou People's Hospital, Yantai, China

**Keywords:** corona virus disease 2019, management, epidemic prevention and control, differential diagnosis, CNS infection

## Abstract

**Importance:** Corona virus disease 2019 (COVID-19) has long latent period, strong infectivity, and non-specific symptoms and signs in the upper respiratory tract. Some initial neurological symptoms appear, including dizziness, headache, seizures, slurred speech, disturbance of consciousness, and limb paralysis among a few COVID-19 patients, which share similar manifestations with central nervous system (CNS) infection. Improving the diagnostic efficiency of suspected CNS infection patients on the basis of preventing and controlling COVID-19 plays a key role in preventing nosocomial and cross infections. This study intends to formulate a hospital emergency management system of fastlane treatment of CNS infection for epidemic prevention and control, aiming at providing references and guidelines for the government and medical institutions to improve the efficiency of treating CNS infection patients in the clinical practice during COVID-19.

**Observations:** This study formulated a framework of a fastlane treatment of CNS infection based on the cooperation of resources and experience, aiming at the key and difficult problems faced by the hospital emergency management system during the COVID-19 outbreak in Changsha, China. The main problem of formulating the hospital emergency management system is efficiently identifying whether CNS infection was caused by severe acute respiratory syndrome coronavirus 2 (SARS-CoV-2). The framework improves the efficiency of diagnosing and treating CNS infections by standardizing the diagnosis and treatment process of patients in emergency observation and strengthening the management of inpatient wards, aiming at assisting medical staff during clinical practice.

**Conclusions and Relevance:** The hospital emergency management system of a fastlane treatment of CNS infection for epidemic prevention and control of the COVID-19 outbreak is a professional and multisystem project, which needs the cooperation of various resources and the experience of clinical leadership.

## Introduction

In December 2019, an outbreak of novel coronavirus pneumonia (NCP) occurred in Wuhan, Hubei province, and rapidly spread to the whole of China, Southeast Asia, Europe, and the Americas. The State Health Committee of the People's Republic of China (PRC) included novel coronavirus pneumonia as a category B infectious disease, but management measures for category A infectious diseases were adopted in China due to its strong infectivity, meaning that new discovered cases are required to be reported within 2 and 6 h in the city and countryside, respectively (1). The World Health Organization (WHO) named the disease as coronavirus disease 2019 (COVID-19), and the International Committee on the Taxonomy of Viruses named the virus as severe acute respiratory syndrome coronavirus 2 (SARS-CoV-2) (2). Major clinical presentations of COVID-19 are initially characterized by fever, fatigue, and cough, which are almost indistinguishable from influenza (3, 4). However, some patients present with non-respiratory symptoms at onset, such as in the nervous, digestive, and cardiovascular systems (5–7). Central nervous system (CNS) infections mostly refers to inflammation caused by pathogens (i.e., bacteria, viruses, and parasites) invading into the CNS, which are mostly manifested as headache, vomiting, convulsions, dysphagia, paresis, anosmia, etc. (8, 9). Patients infected by SARS-CoV-2 can complicate with some neurological symptoms, such as headache, confusion, dizziness, impaired consciousness, altered mental status, ataxia, epilepsy, and skeletal muscle symptoms (10–14), combined with some respiratory symptoms, which could be misdiagnosed as CNS infections caused by other pathogens. These misdiagnoses not only delay the treatment but also increase the exposure risk of other patients and healthcare workers, or even nosocomial exposure risk (9, 15). Therefore, additional attention should be paid to the screening and identification of patients with CNS infection in clinical practice. In this paper, we discuss and summarize the difficulties and countermeasures faced in the fastlane treatment for diagnosis of CNS infections during the COVID-19 outbreak, hoping to provide reference and assistance to other physicians.

## Research Overview on Coronavirus Disease 2019

Coronaviruses (CoVs) are a family of enveloped, unsegmented, positive-sense single-strand RNA viruses that are divided into four genera (α, β, γ, δ), with a diameter of 80–120 nm, and a whole-genome length of 27–32 kb. Currently, CoVs have the largest genome size among RNA viruses (16). CoVs mainly infect the respiratory, digestive, and CNS of humans and livestock (17, 18). CoVs were first isolated from chickens in 1937 and humans in 1965. Electron microscope observations showed that the outer membrane of the virus particles contain obvious and regularly arranged protrusions that resemble the crowns of European monarchs in the Middle Ages. Therefore, they were named as “coronaviruses.” The RNA of CoVs is extremely similar to the mRNA of eukaryotic cells as its 5′ end contains a methylation cap structure, and its 3′ end contains a polyA tail (19). This causes its genomic RNA to skip the RNA–DNA–RNA transcription process, as the RNA can be used as a template for translation. Therefore, there is an extremely high recombination rate between RNAs in CoVs, causing the virus to be prone to mutations (20). The severe acute respiratory syndrome (SARS) outbreak in 2003 and the Middle East respiratory syndrome (MERS) outbreak in 2012 showed that novel CoVs have the potential for animal-to-human and human-to-human transmissions. On December 2019, an outbreak of COVID-19 occurred in Wuhan in the Hubei province and rapidly spread to the whole of China, Southeast Asia, Europe, and the Americas due to its high infectivity, which has attracted global attention. Globally, as of 3:49 pm CEST, July 21, 2020, there have been 14,562,550 confirmed cases of COVID-19, including 607,781 deaths, reported to WHO (21). SARS-CoV-2 is an enveloped positive-sense single-strand RNA virus that contains 29,000 nucleotides and is highly homologous to SARS-like coronaviruses in bats (96.2%) (12). SARS-CoV-2 is the seventh coronavirus to infect humans that has been discovered (the others are HCoV-229E, HCoV-OC43, SARS-CoV, HCoV-NL63, HCoV-HKU1, and MERS-CoV). Its main transmission routes include respiratory droplets and direct contact (12, 22). Other routes such as aerosol, fecal–oral, and mother-to-child transmission still require further studies for confirmation (23, 24). In addition, the human population is generally susceptible, the prognosis is poorer, and the mortality rate is higher in patients with underlying diseases. Compared with SARS, COVID-19 has a longer incubation period, higher infectivity, and patients may not show specific upper respiratory tract signs and symptoms (25). COVID-19 also tends to spread widely, which poses a great challenge in epidemic control. As there is no effective treatment or vaccine at present, the best measures currently are to control sources of infection, early diagnosis, reporting, quarantine, supportive treatment, and timely release of information on the epidemic to prevent unnecessary panic. For individuals, reasonable preventive measures such as good personal hygiene, effective masks, timely indoor ventilation, and avoiding crowded places, can aid in controlling COVID-19.

## Effects and Effector Mechanisms of SARS-CoV-2 on CNS

Coronavirus particles contain an envelope that is composed of a lipid bilayer and mostly glycosylated surface proteins. The envelope surface contains three types of glycoproteins: (1) spike proteins that are key structures for CoV infection and pathogenicity that can recognize and bind to surface receptors on host cells; (2) membrane proteins that are responsible for transmembrane transportation, budding, and release of viruses; and (3) envelope proteins that are responsible for envelope binding. CoVs cannot only invade the respiratory system and immune system but also the nervous, digestive, and cardiovascular systems to cause multisystem damage (26). A study showed that neurotrophic mouse hepatitis virus (MHV) can invade the brain 7 days after intranasal infection to cause encephalitis, meningitis, and demyelination (27). In recent years, SARS-CoV, MERS-CoV, MHV, HCoV-OC43, and other β-coronaviruses have been shown to invade the nervous system. These viruses can infect monocytes and macrophages or vascular endothelial cells to cross the blood–brain barrier and enter the brain. Angiotensin-converting enzyme 2 (ACE2) is widely expressed in monocytes and macrophages as well as alveolar, tracheal, bronchial, and neuronal cells, and is an important target for SARS-CoV invasion (28, 29). SARS-CoV uses the spike protein on its envelope surface to recognize and bind to ACE2 to cause fusion between the virus and host cell, and the genomic RNA of the virus is released into the cytoplasm for replication. SARS-CoV-infected cells can promote T-lymphocyte infiltration, the release of inflammatory factors, and demyelination, resulting in encephalitis and meningitis. SARS-CoV-2 may also have nerve invasion ability since it has the same receptor as SARS-CoV, but further studies are still needed (30, 31).

## Clinical Presentation of Nervous System Infection In COVID-19

Major clinical presentations of COVID-19 include fever, fatigue, and dry cough. However, some patients present with non-respiratory symptoms at onset, such as in the nervous, digestive, and cardiovascular systems. A retrospective study of 214 confirmed COVID-19 patients in Wuhan found that 36.4% showed characteristic nervous system presentation (5). Neurological manifestations can further be subdivided into the CNS symptoms (dizziness, headache, seizures, ataxia, and altered mental status) (32, 33) and peripheral nervous system (anosmia, chemosensory dysfunction, myalgia, weakness, and neuropathy) (33–35) symptomatology. However, it is difficult to distinguish causal relationship from incidental comorbidity. Some other groups reported that nervous system symptoms such as acute cerebrovascular disorder, loss of consciousness, and muscle damage were more frequent and severe in patients with more severe disease course (36). Furthermore, altered mental status, mainly caused by several metabolic and systemic disturbances, has been associated with a worse prognosis (37, 38), and headache and anosmia, which could be an isolated symptom of COVID-19, has been related with a lower probability of death (39, 40). The presence of chronic neurological disorders (CNDs) is also an independent predictor of mortality in hospitalized COVID-19 patients (41). Because the abovementioned CNS symptoms can also appear in patients with hypoxemia, it is impossible to diagnose SARS-CoV-2 infection from clinical manifestations alone. Previous studies indicate the possibility of the neuroinvasive potential of SARS-CoV-2 being the cause of acute respiratory failure, which manifests as respiratory distress and inability to breathe spontaneously (42). Disturbance in smell and taste have become the predominant neurological symptom of COVID-19, which is seen in nearly 80% of patients infected with SARS-CoV-2 (43–47). The loss of smell and taste is found to be more predictive than all other symptoms, such as fatigue, fever, or cough in one recent observation study, which included more than 2 million participants (48). Postviral anosmia has been hypothesized to be the consequence of direct damage to the olfactory sensory neurons (OSNs) responsible for odor detection in the olfactory epithelium (49, 50). However, more evidence in hamster and COVID-19 patients has shown that sustentacular cells are primary targets for SARS-CoV-2 infection rather than OSN (51–53). It still needs further evidence to reveal the mechanisms through which SARS-CoV-2 influences chemical sensing.

## Auxiliary Tests for Nervous System Infections In COVID-19

Blood routine tests show normal or slightly reduced total white blood cell and lymphocyte counts at the early stage of disease. Additionally, studies found lower blood lymphocyte counts among patients with CNS symptoms, which were more frequent and severe in patients with more severe disease course, than those without (5, 12, 34). However, severely ill patients with lower blood lymphocyte counts were more prone to develop neurological complications (54), so we still cannot tell the causal relationship. The D-dimer levels of severe patients were higher than non-severe patients, which may explain why cerebrovascular disease was more frequent and severe in patients with more severe course of COVID-19. Patients with muscular symptoms had higher creatine kinase and lactate dehydrogenase levels than those without muscular symptoms (55). Not every neurologic problem stems from a primary brain injury (56). It is important to rule out systemic causes of neurological symptoms in COVID-19 patients, such as hepatic, renal failure, hypercapnia/hypoxic encephalopathies, coinfections, and treatment-related adverse effects, by blood gas analysis (ABG), liver and renal function tests, blood ammonia, evoked potential (EP), quantitative spectral electroencephalography (EEG) analysis, cerebrospinal fluid (CSF) test, magnetic resonance imaging (MRI), medication and treatment history, etc. (57–60). Previous studies have found some red flags to suspect COVID-19 presence in patients with headache, such as fever, cough, systemic symptoms, prior medical history, some neurologic symptoms, and increased C-reactive protein (61). There were no universal red flags, being the necessary comprehensive evaluation of all of them.

### EEG

A retrospective study showed that the most common indications for EEG among acutely ill COVID-19 patients were new onset encephalopathy (68.2%) and seizure-like events (63.6%), even among patients without prior history of seizures (62). Additionally, sporadic epileptiform discharges (EDs) were present in 40.9% COVID-19 patients, among whom frontal sharp waves (88.9%) were found (62). Triphasic sporadic waves, generalized periodic discharges (GPDs), multifocal periodic discharges (MPDs), and rhythmic delta activity (RDA) were also found among COVID-19 patients with neurological symptoms (63–65). EEG alterations were not specific, which may be related to an underlying morbid status or metabolic and coagulation derangements (65). The EEG of hypercapnia is not specific, with background slowing progressing to discontinuous patterns and burst suppression (56). Although much information may be gleaned from EEG, non-specific EEG findings or abnormalities should not be considered as being specific for COVID-19-related encephalopathy.

### Cranial Imaging

CT or MRI examinations of encephalitis patients may/may not show abnormalities. Preliminary screening with CT can aid in excluding space-occupying lesions. The most frequent MRI findings were signal abnormalities in the medial temporal lobe (43%), non-confluent multifocal white matter hyperintense lesions on FLAIR and diffusion sequences with variable enhancement and associated with hemorrhagic lesions (30%), and extensive and isolated white matter microhemorrhages (24%) (66–68). A consistent MRI finding, multifocal laminar cortical brain lesions were reported in neuro-COVID-19 patients, which were speculated to be related to a possible transient dysregulation of vasomotor reactivity (69, 70). In particular, the cortical involvement may indicate a possible vascular mechanism more shifted toward transient vasoconstriction (69). Additionally, the presence of microbleeds in unusual distribution were found in nine critically ill COVID-19 patients, with a specific predilection for the corpus callosum with or without “blooming artifact” (71, 72). The anterior or posterior limbs of the internal capsule (5/9 patients) and middle cerebellar peduncles (5/9 patients) were other uncommon locations of microbleeds, which could only be depicted in SWI sequence as hypoattenuating foci (71). The study of structural brain abnormalities in 19 non-survivors of COVID-19 performed early (≤24 h) after death found parenchymal brain abnormalities, such as subcortical micro- and macrobleeds (two decedents), non-specific deep white matter changes (one decedent) and cortico-subcortical edematous changes evocative of posterior reversible encephalopathy syndrome (one decedent), and asymmetric olfactory bulbs without downstream olfactory tract abnormalities (four other decedents) (73). Autopsy of COVID-19 patients found that cerebral congestion and edema, and partial neuronal degeneration were present (74, 75), indicating that brain tissue edema may appear in brain MRI among COVID-19 patients.

### CSF

Lumbar puncture and cerebrospinal fluid (CSF) test: in viral encephalitis patients, lumbar puncture pressure is mostly normal or slightly elevated, cerebrospinal fluid white blood cell count is increased but usually <250 × 10^6^/L, and leukocyte subset counts show that lymphocyte percentage is increased. However, neutrophil percentage may increase during early infection. Protein concentration is increased but usually <150 mg/dl, and glucose and chloride are usually normal (75–80). According to a research aiming to evaluate the accuracy and sensitivity of a laboratory-modified CDC-based SARS-CoV-2 N1 and N2 assays across a range of sample types, the N2 target appeared to be most sensitive in SARS-CoV-2 detection in CSF with an LoD of one copy/reaction (81). Previous reports have detected coronavirus nucleic acids such as SARS-CoV, HCoV-OC43, etc., in the CSF of patients with viral encephalitis or multiple sclerosis. More case reports of COVID-19 patients, with positive testing for SARS-CoV-2 by RT-PCR in the CSF, who were also diagnosed with ventriculitis and/or encephalitis, have also followed (82–84). In addition, the presence of SARS-CoV-2 RNA has been detected in the CSF of a patient with clinically proven meningoencephalitis by genome sequencing in Japan (83). Notably, anti-SARS-COV-2 antibodies were detected in the patients' CSF samples (85). Although the specificity remains to be established, it still may constitute a critical diagnostic marker. A definitive diagnosis of central nervous system infection is obtained when the cerebrospinal fluid is positive for SARS-CoV-2 nucleic acid or IgM antibodies. However, most recent studies demonstrate that SARS-CoV-2 is usually not present in the CSF of patients with neurological symptoms, indicating that most neurological symptoms seem to be caused by indirect mechanisms such as systemic critical illness and secondary immune phenomena (86). Like in other virus infections of the brain, a negative PCR test does not exclude the presence of the virus in the brain tissue. Therefore, we should take nasopharyngeal swabs, sputum, lower respiratory tract secretions, blood, CSF, and feces as samples and treat real-time fluorescent RT-PCR, genome sequencing, and serological specific antibody detection as the gold standard for diagnosis, when facing COVID-19-suspected patients.

## Differential Diagnosis of CNS Infection In the COVID-19 Epidemic

CNS infections are one of the most common infections of the nervous system and mostly refers to pathogens (i.e., bacteria, viruses, parasites) invading into the CNS, thereby resulting in inflammation. Viral, purulent, tuberculous, and cryptococcal meningitis are also among these CNS infections. These infections mostly present as chills, fever, and other upper respiratory tract infection symptoms and nervous system symptoms such as headache, vomiting, and convulsions. Severe neurological sequelae will often result if timely treatment is not administered. The different types of encephalitis (meningitis) can be differentiated by season; clinical presentation; physical examination; complete blood count; routine, biochemical, and microbiological tests on the cerebrospinal fluid tests; and imaging tests.

Viral meningitis has become the most common form of meningitis in countries with high rates of immunization coverage as the prevalence of bacterial meningitis is decreasing (87). Enteroviruses (Coxsackie or Echovirus groups) are the most common causes of viral meningitis in all age groups, while parechoviruses are also common in children (88). Viral meningitis most commonly occurs in children with the incidence decreasing with age. Summer and autumn are the high seasons for viral meningitis in temperate climates, which can be present all year round in tropical and subtropical areas (89). Viral meningitis in adults is more prone to present with meningeal symptoms and an elevated CSF protein, while children with viral meningitis are more likely to have fever, respiratory symptoms, and leukocytosis (90). Viral meningitis is characterized by a CSF mononuclear pleocytosis, which may initially be a neutrophilic predominance in the first 24 h of illness and is not a reliable indicator to distinguish viral and bacterial meningitis (91). Meningoencephalitis, myocarditis, and pericarditis are the most common severe complications of enteroviral meningitis, while in children, neurological complications can include acute flaccid paralysis and rhombencephalitis (92, 93).

Due to the lack of clinical findings to help distinguish viral and bacterial meningitis, identifying some predictors of bacterial meningitis has always been research hotspots. The bacterial meningitis score (BMS) was originally developed for children with meningitis, which was comprised of four laboratory predictors including positive Gram stain, CSF protein >80 ml/dl, peripheral absolute neutrophil count >10,000 cells/mm^3^, and CSF absolute neutrophil count >1,000 cells/mm^3^, and one clinical predictor (seizures at or before the presentation) (94). Individual predictors of bacterial rather than viral meningitis have also been found, which include CSF glucose <34 mg/dl, CSF WBC >2,000 cells/mm^3^, CSF neutrophils >1,180, CSF protein >220 mg/dl, and a ratio of CSF to blood glucose <0.23 (95). Additionally, CSF lactate has been indicated to be a great indicator to differentiate bacterial from aseptic meningitis (96, 97). The combination of CSF test and the BMS can increase specificity and sensitivity of distinction (98, 99). Meningococcus, characterized by profound endotoxinemia leading to vasomotor collapse, multiple organ failure, and disseminated intravascular coagulation, is an important and serious bacterial infection. Rapidly enlarging skin and mucosal hemorrhagic lesions (purpura fulminans), and gangrene of digits and limbs caused by arterial thrombi are the clinical hallmarks (100, 101). The predominant feature in children, who do not have adequate immunity against Neisseria meningitides, is septic shock caused by cardiovascular collapse (102).

Viral encephalitis is responsible for high rates of morbidity, permanent neurologic sequelae, and high mortality rates, which usually occur after hematogenic viral dissemination into the CNS (103). Herpesviruses 1 and 2 (HSV-1 and HSV-2), non-polio enterovirus and arboviruses are the most common etiologies. The frequency of specific viruses varies according to season, geographic location, and patient immunological status (104). Host factors and clinical characteristics of infection are important to consider in identifying the cause for viral encephalitis. CSF tests, serology/polymerase chain reaction studies, and neuroimaging are cornerstones of diagnostic evaluation in viral encephalitis (105).

Autoimmune encephalitis is a consequence of inflammation or dysfunction of parts of the brain caused by antibodies against specific brain antigens, usually located in the limbic system, leading to clinical presentations of limbic encephalitis (106). The prevalence of autoimmune encephalitis is increasing, which has surpassed infectious causes of encephalitis in developed countries (107). About 50% of patients with autoimmune encephalitis present or develop fever during the disease course, and most autoimmune encephalitis is associated with CSF lymphocytic pleocytosis (106, 108). Prodromal symptoms including headache and flu-like symptoms occur frequently in autoimmune encephalitis, which may also result in the suspicion of an infectious etiology (109). Fever and inflammation of the CSF are less common than in the infectious causes, but psychiatric symptoms are more frequent (110). Brain MRI can be useful in the differential diagnosis of encephalitis, especially the limbic encephalitis (109).

As the clinical presentation of COVID-19 patients with initial neurological symptoms is highly similar to patients with CNS infection, this not only leads to COVID-19 misdiagnosis and cause them to be invisible spreaders but also tends to lead to nosocomial infection and cross-infection. Therefore, rapidly and correctly identifying patients for admission to the hospital is an important problem currently facing neurologists. Suspected CNS infections caused by SARS-CoV-2 should be differentiated from other viruses, bacteria, and fungi. Extra attention should be paid when collecting the epidemiological history of the patient. Routine, etiological, and serological tests, chest imaging, and plain and enhanced brain MRI tests should be completed. qRT-PCR testing of the cerebrospinal fluid should be carried out as soon as possible in suspected patients to confirm if they have COVID-19. Next-generation sequencing (NGS) and cerebrospinal fluid cultures can be used for etiological tests.

## Treatment of Patients With SARS-CoV-2 CNS Infection In China

Treatment should be carried out in suspected and confirmed cases in designated hospitals with effective isolation and protection conditions. Suspected patients should be quarantined in a single room alone for treatment, while multiple confirmed cases can be treated in the same room. The admission is usually recommended for patients with pneumonia, special risk factors, and/or poor prognostic factors. Critical patients should be admitted to the ICU for treatment as soon as possible.

For patients with symptoms of CNS infections, symptomatic treatments such as dehydration, neuroprotectant, antiepilepsy, and antipsychotic symptoms, should be performed on the basis of general treatment (bed rest, more nutrition, maintaining internal environment stability, monitoring vital signs closely, dynamic monitoring of blood routine, urine routine, C-reactive protein, biochemical indicators, coagulation function, arterial blood gas analysis, chest CT, pressure, and indicators such as cytology, biochemistry, etiology, and autoimmunity-related antibodies of cerebrospinal fluid) and antiviral therapy (remdesivir/α-interferon/lobinavir/ritonavir/ ribavirin/chloroquine phosphate/arbidol) (111). The use of antiviral therapy can be considered for COVID-19 patients with moderate to severe course including pneumonia, those with worsening clinical findings, and those who are likely to progress to severe COVID-19 disease (the elderly, those with chronic diseases, and immunocompromised patients) after the diagnosis or as early as possible (112). Chloroquine (CQ) and hydroxychloroquine (HCQ) have been demonstrated as mechanisms of preventing viral entry and fusion, but these agents should not be used to treat or prevent COVID-19 because of potential serious adverse effects on the cardiovascular, hematologic, hepatic, and renal systems (113–115). Among several potential drugs for the treatment of SARS-CoV-2 infection, remdesivir has shown to be the most promising antiviral therapeutic (116, 117), but recent studies have shown its uncertainties about adverse effects such as nausea, vomiting, hepatic toxicity and rectal hemorrhage, and clinical efficacy (118). Corticosteroids are often used in viral pneumonia, particularly complicated with specific conditions or comorbidities (chronic obstructive pulmonary disease, asthma exacerbation, and septic shock). However, systemic corticosteroids are not currently recommended for the treatment of lung injury associated with SARS-CoV-2 infection (119). The specific mechanism by which steroids act on sustained lung inflammation, as well as the definition of the best drug to use, and even the appropriate treatment duration, are still objectives of ongoing clinical trials (120, 121). There is currently no specific drug targeting SARS-CoV-2 infection in the CNS. Drugs against SARS-CoV-2, which could cross the blood–brain barrier, may be effective theoretically (122). As a result, neurologists should pay attention to the neurological adverse effects of antiviral agents, and the drug interactions related to the use of antiviral agents and antineural symptoms agents, such as antiepileptic drugs and antipsychotic drugs.

For neuro-critical patients, there should be aggressive treatment of complications and underlying disease, prevention of secondary infection, and timely organ function, respiratory, and circulatory support on the basis of symptomatic treatment and microcirculation improvement on the basis of sufficient fluid supplementation. The blood–brain barrier (BBB) hinders most drugs from entering the CNS from the blood stream, leading to the difficulty of delivering drugs to the brain for treatment via the circulatory system (123). Therefore, neurologists prefer antiviral drugs crossing the BBB efficiently and a higher dose for the treatment of CNS infection (124, 125).

For patients with hypertension and suspected CNS infection by SARS-CoV-2, it is theoretically possible that drugs known to increase ACE2 expression, such as ACE inhibitor (ACEI) and angiotensin II receptor blockers (ARBs) could promote SARS-CoV-2 proliferation (126, 127). However, there is insufficient evidence to withdraw ACEIs and ARBs among individuals diagnosed with COVID-19. In fact, most major medical organizations, such as the American Heart Association and European Society of Cardiology supported to maintain ACEI or ARB therapy in all hypertensive patients with COVID-19 (128, 129). Emerging evidence from human studies overwhelmingly suggests that the administration of ACEIs or ARBs does not increase ACE2 expression (130, 131). Furthermore, animal data indicates a potential protective effect of ARBs against COVID-19 pneumonia for the prevention of aggravation of acute lung injury in mice infected with SARS-CoV, which is closely related to SARS-CoV-2 (132, 133). The potential of these agents to facilitate viral disease is still under investigation, irrespective of the purported benefits of ACEIs or ARBs. Management of hypertension with a drug that may reduce inflammation in viral myocarditis (134), which has been considered to be a serious and potentially fatal manifestation of COVID-19 (135, 136), and does not pose a theoretical risk of promoting COVID-19 proliferation, would appear to be a rational strategy to optimize patient outcomes, such as verapamil. Until further data are available, ACEI and ARB medications are still recommended to be continued for the treatment of patients with hypertension and suspected CNS infection by SARS-CoV-2, especially those at high risk.

## Construction of A Hospital Emergency Management System for Fastlane Treatment of CNS Infection During Epidemic Control

In this paper, we propose a basic framework for the treatment of suspected CNS infection patients during an epidemic to increase COVID-19 differential diagnosis efficiency. The aim of this framework is to provide a reference and guide to medical staff, especially neurologists, to improve identification and treatment efficiency in hospitals and reduce the risk of infection exposure in medical staff, which is carried out in Changsha, China.

### Strengthening Staff Control and Standardizing Emergency Department Screening

The hospital emergency management of Xiangya Hospital for fastlane treatment of CNS infection patients could alleviate the fever emergency department, protect health care personnel, and control the cross-infection during the COVID-19 epidemic. We recommend this hospital emergency management for fastlane treatment of CNS infection to hospitals with sustainable supply chains of qualified personal protection equipment and adequate rotating medical staff.

**Figure 1:**
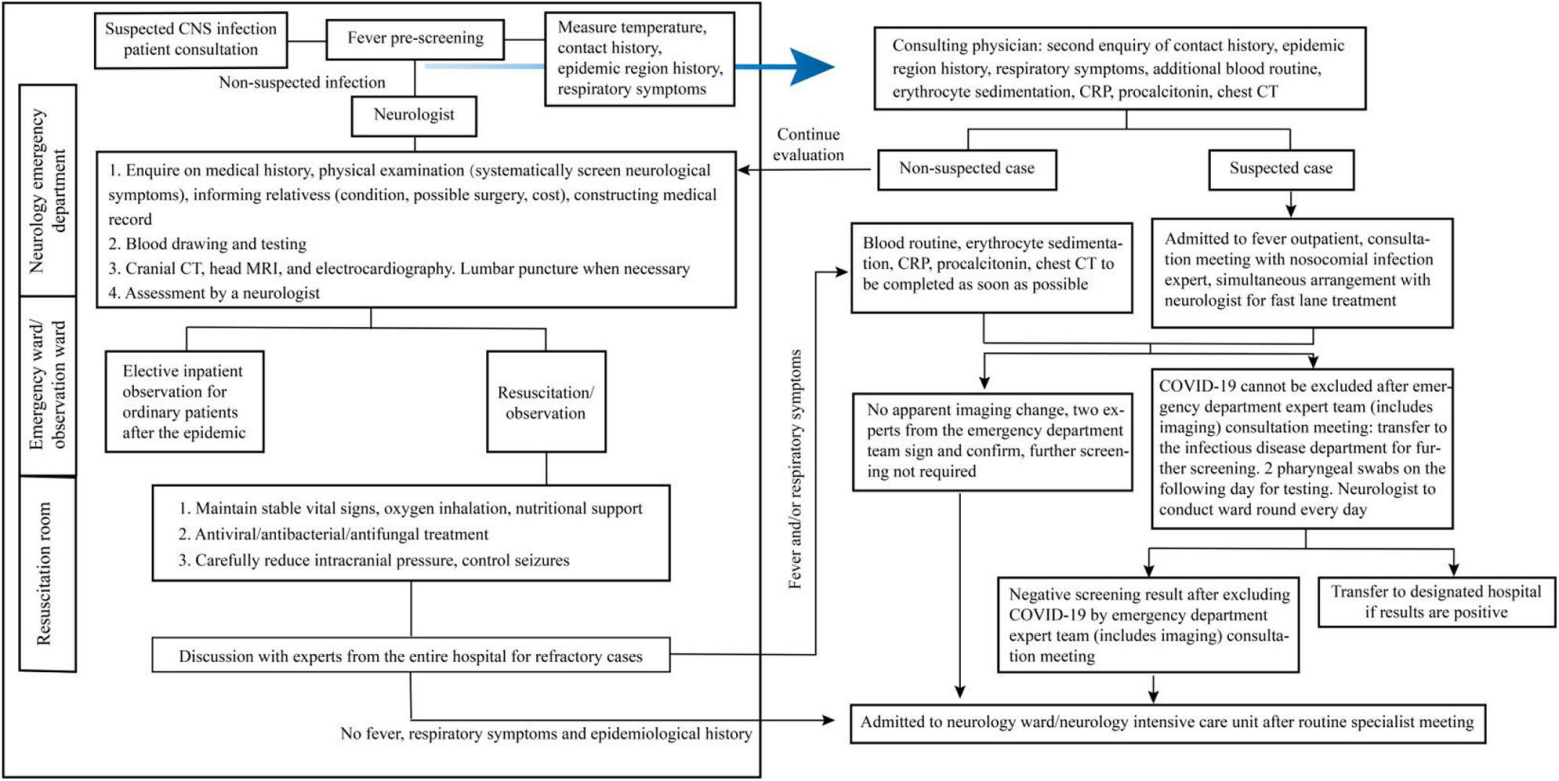
Management flowchart for emergency department admission of suspected central nervous system (CNS) infection patients.

As the initial neurological symptoms of COVID-19 include fever, headache, and epilepsy, it is necessary to strengthen the differentiation of such patients in an emergency department admission. Figure 1 shows the current emergency department admission procedures. Before a suspected CNS infection patient comes into the neurology emergency department, he/she should have a fever prescreening by medical triage staff, ascertainment of history around epidemiology and exposure, and respiratory symptoms. Then the patients with non-suspected COVID-19 infection come into the neurology emergency department; neurologists should follow the left framework for further examination, diagnosis, and treatment. Those suspected COVID-19 infection patients go to the fever isolation emergency for further examination and diagnosis by doctors of the department of infection (following the right framework).

To differentiate CNS infection caused by COVID-19 from potentially lethal central nervous system diseases, some auxiliary tests, such as blood routine test, RT-PCR tests for CSF, and nasopharyngeal swab may be helpful. Herpes simplex virus DNA can be found in CSF, so CSF test may be a helpful way to differentiate CNS infection caused by COVID-19 from herpes simplex encephalitis.

It should be noted that despite the involvement of the central nervous system in COVID-19 infection has been frequently mentioned, only a few cases of encephalitis caused by this infection have been reported. Therefore, in case of signs and symptoms of encephalitis in COVID-19 patients, other causes of damage to the nervous systems should also be carefully considered (137).

### Strengthening Prevention and Control in Medical Staff

#### Conducting Training for the Entire Department

Training on COVID-19 case identification and reporting, epidemiological survey, sampling, laboratory tests, medical treatment and prevention of nosocomial infection, and personal protection should be carried out in the entire department, so that they are equipped with COVID-19 control knowledge, methods, and skills, and understand legal responsibilities and obligations related to epidemic control to achieve early identification, reporting, quarantine, diagnosis, and treatment.

#### Coordinated Deployment of Medical Resources

There should be coordinated deployment of medical resources in the department, rational establishment of a medical echelon, rational shift arrangements, and establishment of an echelon for external assistance. Graded protection is carried out according to the position and zone protection standards and material allocation requirements. Department staff are supervised to strictly comply with the “Medical staff protective gear gowning/degowning procedure,” and suspected medical staff are quarantined and treated in a timely manner. The protective measures for different positions are as follows:

①Primary protection: a. diagnosis, treatment, and nursing of ordinary inpatients; b. triaging of outpatients, triaging and registration of outpatients in the fever outpatient clinic, and timely data reporting; c. cleaning and disinfection of ordinary zones; d. collection of medical waste from ordinary patients; e. ordinary cleaning work; f. testing of ordinary patients by medical technicians; and g. during processing of samples from suspected patients in the laboratory, it is recommended that staff wear masks (N95) and protective goggles (anti-fog) or face shield on the basis of primary protection.

②Secondary protection: (a) staff who diagnose and treat, care, or dispose of medical waste from infected and suspected patients; (b) staff who collect medical waste from infected and suspected patients; and c. staff who carry out cleaning work for suspected or confirmed COVID-19 patients. Work clothes, isolation gown, medical cap, surgical mask with goggles, and surgical gloves are required for secondary protection.

③Tertiary protection: diagnosis and treatment, care, and operation (such as tracheotomy, intubation) in infected or suspected severe patients. On the basis of secondary protection, protective faceshield, waterproof boots, waterproof boot covers, shoe covers, protective clothing plus isolation gown, and double-layer medical surgical gloves are still needed. Surgical mask with goggles and faceshield can be replaced by electric air supply filter respirator.

#### Concern for the Physical and Mental Health of Staff

During a COVID-19 epidemic, clinical staff will face physical, intellectual, and psychological tests, and protecting medical staff is key to overcoming the epidemic. Therefore, rational arrangements of manpower and shifts should be carried out to avoid exhaustion in medical staff. Proactive health monitoring should be carried out based on the characteristics and risk assessment of different positions. Many measures should be adopted to protect the physical and mental health of medical staff and help the families of frontline medical staff. A detailed psychological intervention plan was developed by The Second Xiangya Hospital, which mainly covered the following three areas: building a psychological intervention medical team, which provided online courses to guide medical staff to deal with common psychological problems; a psychological assistance hotline team, which provided guidance and supervision to solve psychological problems; and psychological interventions, which provided various group activities to release stress (138).

## Summary

Patients with SARS-CoV-2 infection have different neurological presentations, such as dizziness, headache, seizures, ataxia, altered mental status, anosmia, chemosensory dysfunction, myalgia, weakness, and neuropathy. Improving the identification efficiency for COVID-19 during the admission of CNS infection patients during the COVID-19 epidemic is an important area and challenge in prevention work in the neurology department. This review summarized the mechanism of SARS-CoV-2 on the CNS, clinical presentations, auxiliary tests, differential diagnosis, and treatment of patients with neurological symptoms of SARS-CoV-2 infection, as well as a hospital emergency management system of fastlane treatment of CNS infection for epidemic prevention and control, aiming at providing references and guidelines for the government and medical institutions to improve the efficiency of treating CNS infection patients in the clinical practice during COVID-19. CoV is a serious global health threat. Due to climate and ecological changes, human–animal interactions continue to increase. The emergence and outbreak of SARS-CoV-2 suggest that CoV outbreaks will be unavoidable in the future. Therefore, development of effective treatments and vaccines for CoVs is urgently required. At present, there are no specific prophylactic drugs for SARS-CoV-2. Therefore, strengthening identification during clinical treatment is important in preventing nosocomial and cross-infections.

## Author Contributions

All authors listed have made a substantial, direct and intellectual contribution to the work, and approved it for publication.

## Conflict of Interest

The authors declare that the research was conducted in the absence of any commercial or financial relationships that could be construed as a potential conflict of interest.
